# The Impact of Vapor Blockage on the Outflow Rate of Screen Channel Liquid Acquisition Devices

**DOI:** 10.3390/mi13020322

**Published:** 2022-02-18

**Authors:** Jian Li, Yuan Ma, Yanzhong Li, Bin Wang, Hui Zang

**Affiliations:** 1 Institute of Refrigerating and Cryogenic Engineering, School of Energy and Power Engineering, Xi’an Jiaotong University, Xi’an 710049, China; lijian144.110.11@stu.xjtu.edu.cn (J.L.); yzli-epe@xjtu.edu.cn (Y.L.); 2 State Key Laboratory of Technologies in Space Cryogenic Propellants, Beijing 100028, China; 3 Aerospace System Engineering Shanghai, Shanghai 201109, China; wangbin_cryo@163.com (B.W.); zanghui805@163.com (H.Z.)

**Keywords:** screen channel liquid acquisition device (LAD), cryogenic propellants, vapor blockage, outflow rate, robustness

## Abstract

The outflow rate of screen channel liquid acquisition devices (LADs) is a key indicator of the liquid acquisition capacity, but would be decreased when a portion of its screen is blocked by the vapor. So far, the quantitative research about the consequent loss of outflow rate seems not enough, though it is important and inevitable. In this paper, a modified model by introducing an “available rate” to describe the blocked degree is established to analyze and compare the cases with and without vapor blockage. We found that the loss of outflow rate is mainly decided by the total area of the blocked screen, while the distribution of blockage position barely has any effects. Besides, a “characteristic curve” is proposed to describe the robustness of LAD against blockage (i.e., loss rate of outflow velocity versus total area of the blocked screen). Higher driving pressure, coarser mesh of screen, and higher ratio of length to height of the channel would bring about greater robustness.

## 1. Introduction

In space flight missions, the pure liquid-phase propellant is necessary when the engines restart or when the system transports propellant from one tank to another [1]. However, in the microgravity environment, acquiring pure liquid from the liquid–vapor mixture in the tank is not easy. The fluid motion at reduced gravity may be dominated by the surface tension of the fluid and other forces, which make it nearly impossible to predict how liquid and vapor distribute in the propellant tank, let alone to ensure enough pure liquid to cover the tank outlet. To address this difficulty, researchers invented liquid acquisition devices (LADs), which come in various forms and fall into two main categories: partial retention device and total communication device [2]. The former retains a part of liquid around the outlet for the engine to restart but is generally applicable for the systems that only require thrust in one single direction. The latter aims to make a full communication between the outlet and the liquid, and it can give supply continuous outflow under any thrust directions.

**Figure 1:**
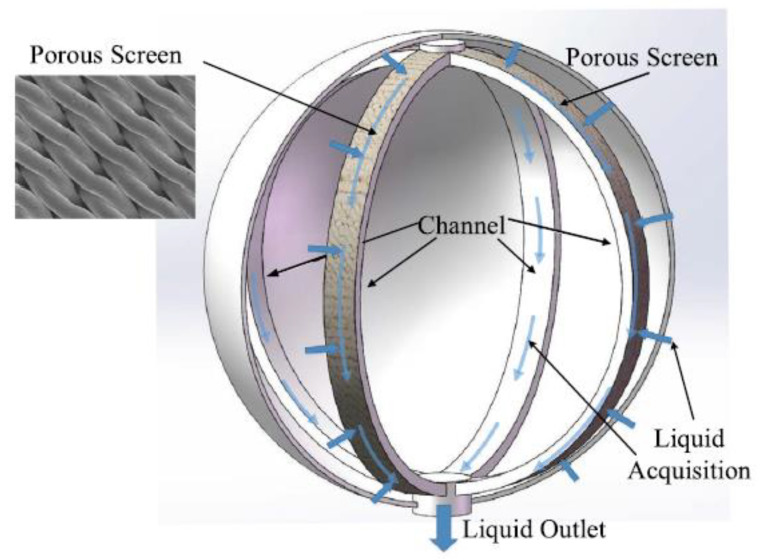
Schematic diagram of a typical screen channel LAD.

**Figure 2:**
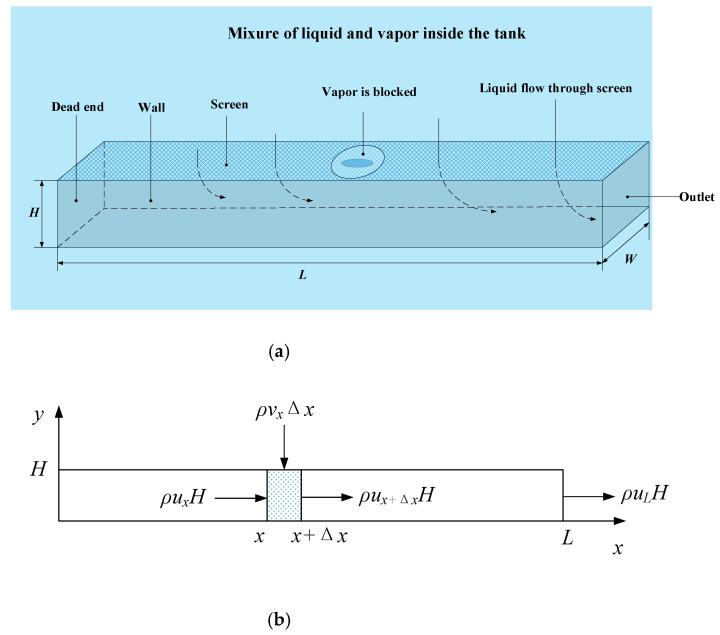

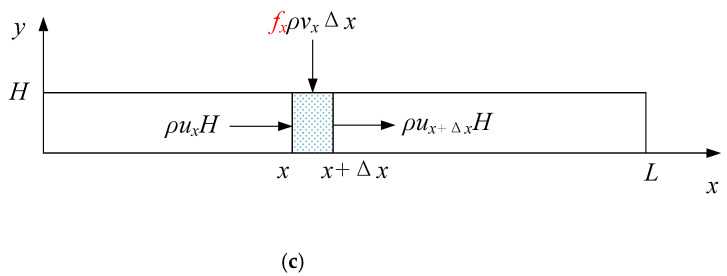
Schematic diagram of a single channel. (**a**) 3-D diagram; (**b**) Diagram for basic outflow model; (**c**) Diagram for the extended model.

Among the total communication devices, screen channel LADs are popular in-flight missions that involve cryogenic propellants. This device is installed inside the tank (see the schematic in Figure 1); one typical structure consists of four channels that run almost the full length of the tank. Each channel is a rectangular cross-section pipe with four walls: three solid metal walls and a porous screen wall (see Figure 2a). The porous screen is woven by metal wires, and those woven by the method of Dutch Twill weave (DTW) [3] proved to perform the best for cryogenic liquids. Because the surface tension of fluid leads the liquid to distribute along the inner wall of the tank under microgravity conditions, channels generally turn their porous screen wall toward the inner wall of the tank to contact more liquid.

The liquid acquisition process is as follows: the liquid that contacts the porous screen will be wicked [4,5,6] by the tortuous micro flow path formed between wires of the screen so that the whole screen would be wet; when the system pressurizes the ullage of tank, the surface tension of the liquid that has been filled within the screen pores will stop the vapor from penetrating the screen, while the liquid will be driven into the channel through the screen by the pressure difference effect; eventually, the liquid flow along the channel and towards the outlet while the vapor is retained in the tank (see Figure 2a), and the pure liquid-phase propellant is thus acquired.

During this process, two vital parameters of the screen are involved: bubble point pressure (BBP) and flow-through-screen (FTS) pressure drop. The so-called BBP is a critical pressure difference between two sides of a screen, if the pressure difference (i.e., pressure of gas side minus that of liquid side) exceeds this critical value, gas will break through the screen and cause device failure. BBP is determined by the surface tension of the working fluid, the structure of the screen, and the contact angle between the liquid and the material of the screen. The exact value of BBP for various screens and fluid should be measured experimentally before being applied by LAD designers, although there has been an existing simplified prediction model [7]:

(1) $$\Delta P_{\mathrm{BP}}=\frac{4\sigma \cos\theta }{D_{P}},$$

where *σ* denotes the surface tension of the fluid, *θ* represents the contact angle between the fluid and the material of the screen wire, and *D*_p_ indicates the “effective diameter” of the pore (the complex geometry of pore between screen wires is simplified into a cylindrical hole).

FTS pressure drop is the local pressure loss due to the flow resistance of the screen. A commonly used prediction model is given by Armour and Canno [8],

(2) $$\Delta P_{\mathrm{FTS}}=C_{l}\mu v+C_{t}\rho v^{2},$$

where *C_l_* and *C_t_* are viscous resistance coefficient and inertial resistance coefficient respectively, which depend only on the structure of the screen. *μ* is the viscosity of the fluid, *ρ* is the density of the fluid, and *v* denotes the local velocity perpendicular to the screen (injection velocity). During the process of liquid acquisition, a different part of the screen undergoes various local injection velocities and the FTS pressure drop changes along the channel in consequence; once the FTS pressure drop exceeds BBP at one or more positions, the device will be at the risk of bubble breaking through failure. Hence, the designers for screen LAD should make sure the distribution of the FTS pressure drop is safe enough under the demand outflow rate.

To achieve this goal, researchers have conducted a lot of work to study how injection and FTS pressure drop are distributed during the liquid acquisition process. Many early researches [9,10,11] and even some latter researches [12,13,14] have held the simple assumption that the injection velocity is constant along the channel, and the results based on this assumption showed inevitable deviations from reality. This ideal assumption was gradually given up in the work by Quintard [15] and Galowin et al. [16], where the effect of viscous resistance (the first term of the right side in Equation (2)) was taken into account. However, the modified model could only have a good precision at low injection velocity conditions. Until the work by Hartwig et al. [17], the second term (inertial resistance term) of Equation (2) was introduced and the model was then capable of predicting the case with high injection velocity. However, this model is not so accurate because it applied an unnecessary boundary condition (i.e., no-slip condition at the dead end of the channel). As a result, Darr et al. [18] updated the model by abandoning this inappropriate boundary condition and then got the experiment-validated prediction results. The work of [18] demonstrated that both FTS pressure drop and injection velocity increased along the axis direction of the channel. Besides, some CFD simulations [19,20,21] were conducted to obtain the detailed information about the FTS pressure drop and injection velocity at specific conditions, but a further theoretical explanation was not given.

Overall, previous researchers focus mainly on the cases where the screen is fully submerged in liquid. However, when a part of the screen is blocked by vapor (see Figure 2a), the outflow rate would deviate from the design value. So far, research into how blockage affects the device performance seems to be inadequate. In view of this, this paper developed a mathematical model to analyze the impact of vapor blockage on the outflow rate of the channel. We characterized the blockages by two factors (i.e., the position where the blockages occur, and the area of blocked screen), and then investigated their impact respectively. Finally, a “characteristic curve” was defined to reveal the robustness of the device, and the three influential factors that affect the shape of the characteristic curve was also analyzed.

## 2. Materials and Methods

Analysis about the vapor-blocked case should be based on a good understanding of the normal case without blockage. Consequently, the basic outflow model would be introduced.

### 2.1. Basic Model

Consider a rectangular channel with length *L*, width *W*, and height *H*; the coordinate origin is located at the dead end of the channel (see Figure 2a). During the outflow process, the liquid in the tank flows through the screen (with the injection velocity v, while undergoing a FTS pressure drop $\Delta P_{\mathrm{FTS}}$), into the channel, and then along the channel to the outlet. The average transverse velocity of the channel cross-section (represented by u), as well as v and $\Delta P_{\mathrm{FTS}}$, varies along the *x* direction, so the subscripts, such as x, x+Δx and *L*, are used to mark the *x* coordinates of the variable.

To simplify the model, three assumptions were applied:

1. Flow in the width direction (i.e., *z*-direction) can be neglected.
2. The gravity is negligible because the device works in microgravity conditions.
3. The pressure loss due to wall friction is negligible. The reasonability of this assumption has been verified in [18] that the FTS pressure drop dominates over the frictional pressure drop.

The pressure outside the channel, $P_{\mathrm{ullage}}$ (i.e., the pressure of the ullage of the tank), is constant along the length. Under steady-flow conditions, the pressure difference between the outlet and the ullage has been established, and this pressure difference (i.e., $P_{\mathrm{ullage}}-P_{\mathrm{outlet}}$) is defined as the driving pressure ($\Delta P_{d}$) in this paper. Driven by $\Delta P_{d}$, liquid in the tank flows through the screen into the channel, undergoing different local $\Delta P_{\mathrm{FTS}}$. The consequent difference of pressure at every cross-section inside the channel (i.e., the $P_{\mathrm{ullage}}$ minus the local $\Delta P_{\mathrm{FTS}}$) drives the liquid flowing along the length direction from the dead end to the outlet.

Because of assumption (1), the flow is reduced to a 2-D process, as shown in Figure 2b. Considering a volume element with the length of Δx, liquid with the density of *ρ* enters from two directions (see Figure 2b): the portion entering from above, expressed as $\rho v_{x}\Delta x$, is collected by the exact screen of this element; while the portion entering from the left, expressed as $\rho u_{x}H$, is acquired by the screen of the upper stream and is accumulated along the channel. The confluence of these two portions flows out of the volume element toward the lower stream, at the velocity of $u_{x+\Delta x}$. According to the principle of conservation of mass:

(3) $$\rho u_{x}H+\rho v_{x}\Delta x=\rho u_{x+\Delta x}H.$$

We apply the Taylor expansion to $u_{x+\Delta x}$, with the second-order small qualities being neglected, and then divide both sides of Equation (3) by ρΔx:

(4) $$v_{x}=H\frac{du_{x}}{dx}.$$

The momentum conservation in the *x*-direction is expressed as:

(5) $$\Delta t\rho u_{x}H\left(u_{x+\Delta x}-u_{x}\right)+\Delta t\rho v_{x}\Delta x\left(u_{x+\Delta x}-0\right)=\left(P_{x}-P_{x+\Delta x}\right)H\Delta t.$$

which means that during a very short time of Δt, the liquid that entered the volume element got an increase in momentum (i.e., the left side of this equation) caused by the pressure difference between the left and the right boundary of the element (i.e., the right side of this equation). The pressure of the two boundaries are as follows:

(6) $$P_{x}=P_{\mathrm{ullage}}-\Delta P_{\mathrm{FTS},x}$$

(7) $$P_{x+\Delta x}=P_{\mathrm{ullage}}-\Delta P_{\mathrm{FTS},x+\Delta x}$$

According to Equation (2), the local FTS pressure drops of the left and right sides of the volume are

(8) $$\Delta P_{\mathrm{FTS},x}=C_{l}\mu v_{x}+C_{t}\rho v_{x}^{2}$$

and

(9) $$\Delta P_{\mathrm{FTS},x+\Delta x}=C_{l}\mu v_{x+\Delta x}+C_{t}\rho v_{x+\Delta x}^{2},$$

respectively.

Substitute Equations (4), (6)–(9) into Equation (5), and then apply Taylor expansion to $u_{x+\Delta x}$ and $v_{x+\Delta x}$*,* with the higher-order small qualities being neglected. After being divided by ΔxΔt, it can be expressed as

(10) $$2\rho u_{x}\frac{du_{x}}{dx}=HC_{l}\mu \frac{d^{2}u_{x}}{dx^{2}}+2C_{t}\rho H^{2}\frac{d^{2}u_{x}}{dx^{2}}\frac{du_{x}}{dx}.$$

To solve this second-order nonlinear ordinary differential equation, two boundary conditions are needed. The first one is given by the fact that *u* at the dead end (*x* = 0) equals zero, while the other one should be chosen according to what kind of information is given:

(a) the channel’s total outflow mass rate ${\dot{q}}_{m}$;
(b) the driving pressure, or $\Delta P_{d}$.

About information (a), the average transverse velocity at the cross-section of the outlet is

(11) $$u_{L}=\frac{{\dot{q}}_{m}}{WH\rho }.$$

Hence, one set of boundary conditions has the form as

(12) $$\{\begin{array}{c} u_{x}=0,\;\;\;\;\;\;\;\;x=0\\ u_{x}=\frac{{\dot{q}}_{m}}{WH\rho },\;\;x=L \end{array}$$

For information (b), since $\Delta P_{d}$ is equal to the FTS pressure drop at the outlet (i.e., $\Delta P_{\mathrm{FTS},L}$), considering Equation (2), the injection velocity at the outlet of the channel is

(13) $$v_{L}=\frac{-C_{l}\mu +\sqrt{{\left(C_{l}\mu \right)}^{2}+4C_{t}\Delta P_{d}}}{2C_{t}\rho }.$$

taking Equation (4) into account, while *x* = *L*,

(14) $$\frac{du_{x}}{dx}=\frac{-C_{l}\mu +\sqrt{{\left(C_{l}\mu \right)}^{2}+4C_{t}\Delta P_{d}}}{2HC_{t}\rho }.$$

Consequently, the other set of boundary conditions (i.e., driving pressure boundary condition) is

(15) $$\{\begin{array}{c} u_{x}=0,\;\;\;\;\;\;\;\;\;\;\;\;\;\;\;\;\;\;\;\;\;\;\;\;\;\;x=0\\ \frac{du_{x}}{dx}=\frac{-C_{l}\mu +\sqrt{{\left(C_{l}\mu \right)}^{2}+4C_{t}\Delta P_{d}}}{2HC_{t}\rho },\;\;x=L \end{array}$$

The shooting method, as well as the variable-step fourth-fifth order Runge–Kutta method and string section method, was applied to solve Equation (10). Boundary conditions should be selected between Equations (12) and (15) for different given variables (${\dot{q}}_{m}$ or $\Delta P_{d}$). After getting $u_{x}$, $v_{x}$ and $\Delta P_{\mathrm{FTS},x}$ could be solved out from Equations (4) and (8), respectively.

### 2.2. Model Validation

**Figure 3:**
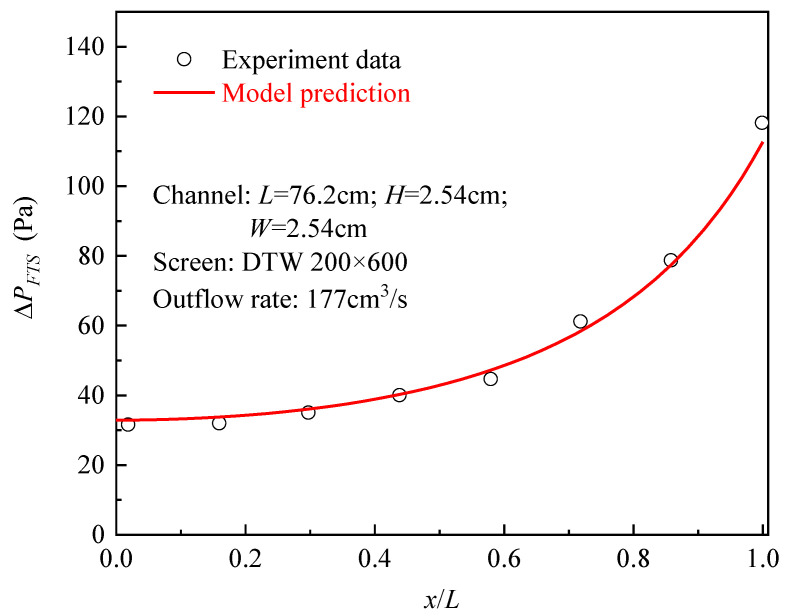
Comparison between model prediction and historical experimental data.

To validate the model, a set of experiment data from [18] is compared with the model prediction about the distribution of FTS pressure drop. The experimenters selected room-temperature distilled water as the working fluid and then conducted a horizontal outflow test using a channel (equipped with the screen of DTW200 × 600) with *L* of 76.2 cm, *H* of 2.54 cm, and *W* of 2.54 cm. Under the flow rate of 177 cm^3^/s, they measured the local $\Delta P_{\mathrm{FTS}}$ at eight points along the length direction. The model prediction and the experiment data show good fitness, with an average error of 4.8%. (Figure 3).

## 3. Results and Discussion

### 3.1. Influential Factors of the Outflow Rate for Cases without Blockage

According to Equation (11), the outflow rate ${\dot{q}}_{m}$ could be denoted by the transverse velocity at the outlet $u_{L}$. For a given working liquid, three factors, including the driving pressure $\Delta P_{d}$ the geometric parameters and the screen structure, would affect the total outflow rate of the channel, according to Equation (10) and the driving pressure boundary condition in Equation (15). The mechanism of these three influential factors would be discussed respectively below. Without the loss of generality, saturated liquid hydrogen at the pressure of 100 kPa is chosen as the working fluid, with a density of 70.9 kg/m^3^ and viscosity of 13.54 × 10^−6^ Pa·s.

#### 3.1.1. Influence of Geometric Parameters

**Figure 4:**
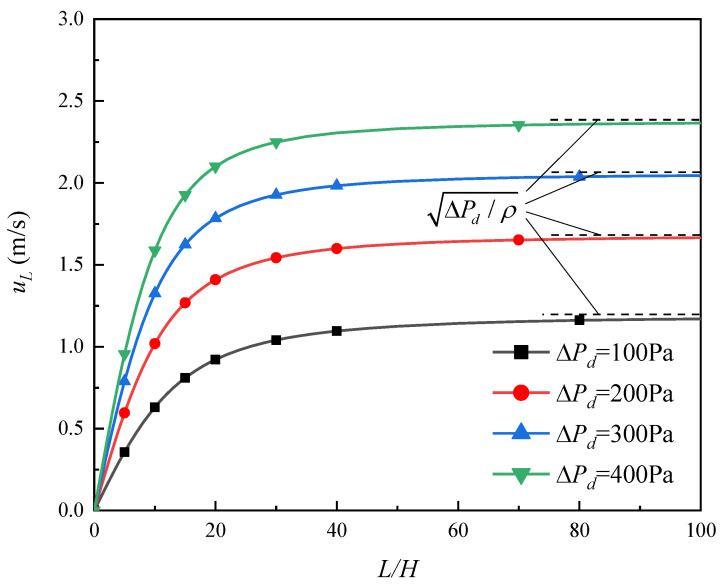
Outflow velocity versus *L/H* at different driving pressures.

For the 2-D flow process discussed here, only *L* and *H* exert influence. In order to learn how *L* and *H* affect $u_{L}$, we investigate 15 channels with different sizes under the same conditions, the calculated $u_{L}$ are listed in Table 1. All of these channels are being driven by the same $\Delta P_{d}$ of 519 Pa and are equipped with the same screen of DTW325 × 2300. The values in every column of Table 1 are equivalent to each other, which indicates that $u_{L}$ is a function of the ratio of *L* to *H* (i.e., *L/H*), rather than of *L* or *H* individually. Due to this finding, the relationship between $u_{L}$ and *L/H* at different $\Delta P_{d}$ was further investigated, with the results shown in Figure 4 (where all the channels are equipped with the screen of DTW325 × 2300).

Figure 4 shows that when *L/H* is at a lower range, the outflow velocity $u_{L}$ increasing nearly proportionally with the increasing *L/H*; but while *L/H* gets a range that is high enough, $u_{L}$ gradually approaches its upper value of $\sqrt{\Delta P_{d}/\rho }$ (which will be interpreted in Section 3.1.2). In fact, a constant average injection velocity of the total screen would bring about the situation that $u_{L}$ increases proportionally with *L/H*. Consequently, the declining growth trend of $u_{L}$ in Figure 4 means the decreasing average injection velocity of the total screen with the increasing *L/H* (especially when it exceeds the value of 40).

In summary, as *L/H* increases, $u_{L}$ increases rapidly first and then levels off to approach the upper limit value of $\sqrt{\Delta P_{d}/\rho }$, thus further elevating *L/H* could not bring about a significant increase of $u_{L}$.

#### 3.1.2. Influence of Driving Pressure

The driving pressure $\Delta P_{d}$ would decide the upper limit of outflow velocity (i.e., $\sqrt{\Delta P_{d}/\rho }$) for given liquid, as shown in Figure 4. To interpret, the momentum theorem at the length direction is applied to the whole channel:

(16) $$WH\left(P_{\mathrm{ullage}}-\Delta P_{\mathrm{FTS},0}\right)-WH\left(P_{\mathrm{ullage}}-\Delta P_{\mathrm{FTS},L}\right)=WH\rho u_{L}\left(u_{L}-0\right),$$

where the left side represents the driving force, and the right side indicates the change of momentum per unit time. Then taking into account the fact that $\Delta P_{FTS,0}>0$ and $\Delta P_{d}=\Delta P_{\mathrm{FTS},L}$, the relation of

(17) $$u_{L}<\sqrt{\Delta P_{d}/\rho }$$

could be obtained, which interprets the upper value.

Besides, during the outflow process, the FTS pressure drop increases along the *x*-direction and gets its maximum at the outlet (see Figure 3). Since the FTS pressure drop at any location must not exceed the bubble point pressure $\Delta P_{\mathrm{BP}}$, it should be assured that $\Delta P_{\mathrm{FTS},L}\leq \Delta P_{\mathrm{BP}}$ (i.e., $\Delta P_{d}\leq \Delta P_{\mathrm{BP}}$). Therefore, the outlet is always the riskiest zone where bubble breaking may occur, and a channel could get its maximum allowable outflow velocity (critical $u_{L}$) only when the $\Delta P_{d}$ is equal to $\Delta P_{\mathrm{BP}}$. Thus, for given liquid and screen type, the critical $u_{L}$ should be $\sqrt{\Delta P_{\mathrm{BP}}/\rho }$.

#### 3.1.3. Influence of Screen Mesh

The screen exert influence on the outflow rate by two factors: bubble pressure $\Delta P_{\mathrm{BP}}$, and the resistance coefficients (i.e., $C_{l}$ and $C_{t}$). The $\Delta P_{\mathrm{BP}}$ decides the upper limit of driving pressure and consequently the maximum allowable outflow velocity, while $C_{l}$ and $C_{t}$ significantly affects the pressure drop. The higher $\Delta P_{\mathrm{BP}}$ and lower $C_{l}$ and $C_{t}$ are always preferred.

When selecting the screen, the weave type and fineness of mesh are to be considered. Since the popularity of the DTW screen among LADs, the choice should be made merely between finer mesh or coarser mesh. Finer mesh screen often possesses higher $\Delta P_{BP}$ but higher resistance coefficients, while coarser mesh screen is the opposite. Therefore, a comprehensive comparison should be made.

**Figure 5:**
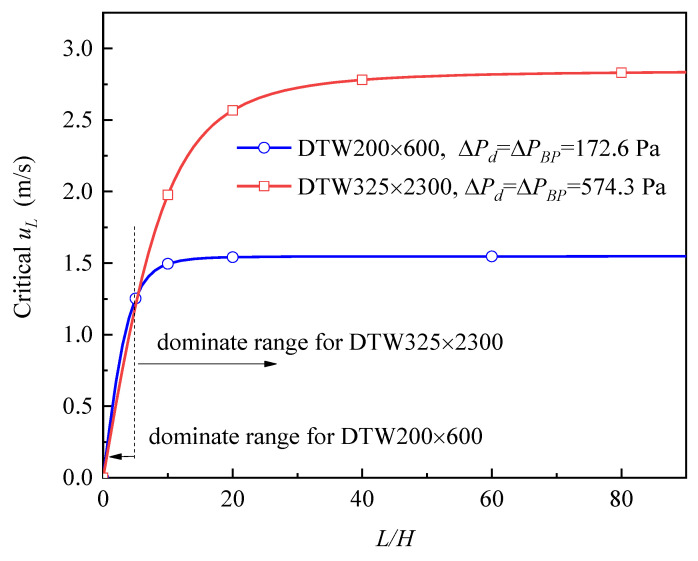
Critical outflow velocity versus the *L/H* of the channels.

DTW200 × 600 and DTW325 × 2300 are chosen as the representative of coarse and fine mesh screens, respectively. Channels equipped with the two screens are investigated under a wide range of *L/H*, respectively. Since the comparison is conducted with the critical $u_{L}$ as the evaluation criterion, at each *L/H*, the channel is driven by its own $\Delta P_{\mathrm{BP}}$ (i.e., 172.6 Pa for DTW200 × 600 and 574.3 Pa for DTW325 × 2300) [22]. We can see from Figure 5 that the coarse mesh screen enables the channel to get higher critical $u_{L}$ when *L/H* is lower than 6.2, but things are opposite when *L/H* is higher in this case. To interpret, higher outflow velocity can be brought about by both higher driving pressure and lower resistance; in the dominant range for DTW200 × 600, the lower resistance contributes more to the higher outflow velocity, while in the dominant range for DTW325 × 2300, the higher driving pressure (allowed by the higher $\Delta P_{\mathrm{BP}}$) contributes more.

In short, the advantages of fine mesh screen (i.e., higher $\Delta P_{\mathrm{BP}}$) and coarse mesh screen (i.e., lower resistance) should be traded off, with coarser mesh being suitable for the channel with lower *L/H* while finer mesh is the opposite.

### 3.2. Analysis of the Case with Vapor Blockage

The blockage is characterized by two factors: the location where the blockage occurs, the total area of the blocked zone. To enable the description of the vapor blockage case, the model needs to be extended.

#### 3.2.1. Extended Model for the Case with Vapor Blockage

The rectangular channel in Figure 2 dashed as the research object, and the variables to be solved are still average transverse velocity of channel cross-section ($u_{x}$), injection velocity ($v_{x}$), and FTS pressure drop ($\Delta P_{FTS,x}$), which change along the *x* direction.

**Figure 6:**
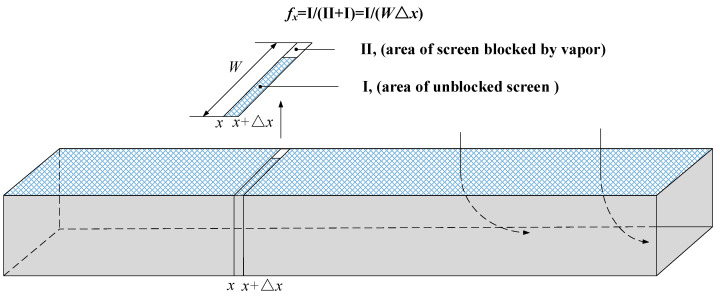
Definition of available rate *f_x_*.

In order to flexibly describe the variable situation of vapor blockage along the channel, we introduce a correction factor *f_x_* named “available rate”. It means the percentage of the screen that is not blocked by vapor in the neighborhood of *x* (see Figure 6). The value of *f_x_* is between 0 and 1 to reflect the blocked degree between non and complete blocking. The blockage position can be expressed by adjusting the location of *f_x_* along the *x*-axis while the total percentage of the blocked area can be represented by the value of $1-\frac{1}{L}\int_{0}^{L}f_{x}dx$. It should be noted that $f_{x}$ does not include the information about how the vapor distributes along the width direction; thus it is only a function of *x*.

Selecting a control volume element from the channel (see Figure 2c and Figure 6), the conservation of mass could be expressed as

(18) $$\rho u_{x}H+f_{x}\rho v_{x}\Delta x=\rho u_{x+\Delta x}H,$$

which means the equality between the liquid that flows into (from the upstream of the channel or through the screen) and out of the element. Doing Taylor expansion of Equation (18), while omitting the higher-order small quality, mass conservation then can be expressed as

(19) $$f_{x}v_{x}=H\frac{du_{x}}{dx},$$

While applying the momentum theorem to this control volume element, pressure drop due to the friction between liquid and the inner wall of the channel could be neglected, according to assumption 3. Thus, for the control element, the change of theorem in the *x*-direction is driven only by the pressure difference:

(20) $$\rho u_{x}H\left(u_{x+\Delta x}-u_{x}\right)+f_{x}\rho v_{x}\Delta x\left(u_{x+\Delta x}-0\right)=H\left(P_{x}-P_{x+\Delta x}\right),$$

Taking into account the definition of FTS pressure drop, Equation (20) then evolves to the following form, after Equations (6)–(9) are substituted into it.

(21) $$2\rho u_{x}\frac{du_{x}}{dx}=HC_{l}\mu \frac{dv_{x}}{dx}+2C_{t}\rho H^{2}v_{x}\frac{dv_{x}}{dx}$$

Equation (19), Equation (21), and the expression of $f_{x}$ form the equation group that governs the outflow process. To solve the set of equations, two boundary conditions are also needed and the first one can still be obtained by the fact that *u*_(*x*=0)_ = 0. As for the second boundary condition, the driving pressure boundary condition below is determined jointly by Equations (13) and (19):

(22) $${\frac{du_{x}}{dx}|}_{x=L}=\frac{-C_{l}\mu +\sqrt{{\left(C_{l}\mu \right)}^{2}+4C_{t}\Delta P_{d}}}{2HC_{t}\rho }{f_{x}|}_{x=L}.$$

Thus, the boundary conditions are

(23) $$\{\begin{array}{c} u_{x}=0,\;\;\;\;\;\;\;\;\;\;\;\;\;\;\;\;\;\;\;\;\;\;\;\;\;\;\;\;x=0\\ \frac{du_{x}}{dx}=\frac{-C_{l}\mu +\sqrt{{\left(C_{l}\mu \right)}^{2}+4C_{t}\Delta P_{d}}}{2HC_{t}\rho }f_{x},\;\;x=L \end{array}$$

Since the distribution of $f_{x}$ along the *x*-direction is a known input parameter, the process of solving the governing equations should be as follows: (1) get the second-order nonlinear differential equation about $u_{x}$ by substituting Equation (19) and the expression of $f_{x}$ into Equation (21); (2) solve $u_{x}$ from this equation using shooting method while applying the boundary conditions of Equation (23); (3) solve $v_{x}$ and $\Delta P_{\mathrm{FTS},x}$ from Equation (19) and Equation (8), respectively.

#### 3.2.2. Impact of Blockage Position

To research the impact of blockage position on outflow velocity $u_{L}$, the other influential factor should be fixed. This is equivalent to exploring how the distribution of $f_{x}$ affects $u_{L}$ under fixed $1-\frac{1}{L}\int_{0}^{L}f_{x}dx$.

Without loss of generality, we fix the value of $1-\frac{1}{L}\int_{0}^{L}f_{x}dx$ as 0.5, and then investigate a channel (30 cm long, 1 cm high, and 1 cm wide) equipped with the screen of DTW325 × 2300. Saturated liquid hydrogen at the temperature of 20 K is still chosen as the working fluid. The device keeps the constant driving pressure of 574 Pa while adjusting the available rate $f_{x}$ in different ways.

**Figure 7:**
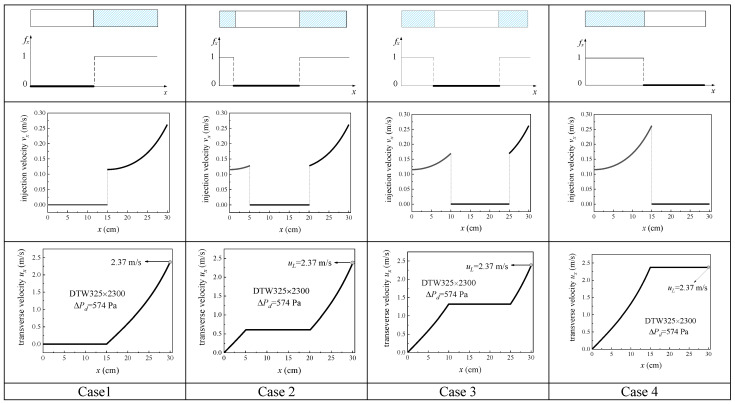
Comparison of cases with a single bubble at four different locations.

First, we start from the simplest cases: all the blockages are concentrated on a section of the screen (like a “large bubble”). To simulate this distribution mode of the blockage area, we let a segment of $f_{x}$ be 0 while the rest of this segment to be 1 (see case1~case4 in Figure 7, in which the blocked area is marked by the white part while the available part of the screen is denoted by the blue part). When this blocked area moves from upstream to downstream of the channel, the injection velocity $v_{x}$ and transverse velocity $u_{x}$ will show correspondingly various distribution patterns (see Figure 7). In the blocked zone, $v_{x}$ and $u_{x}$ remain unchanged, while in the available area, both $v_{x}$ and $u_{x}$ increase with *x* coordinate. Interestingly, all $u_{x}$ in the four cases get the same value of 2.37 m/s at the outlet of the channel, which hints that the blockage position of a single bubble does not affect the outflow rate.

**Figure 8:**
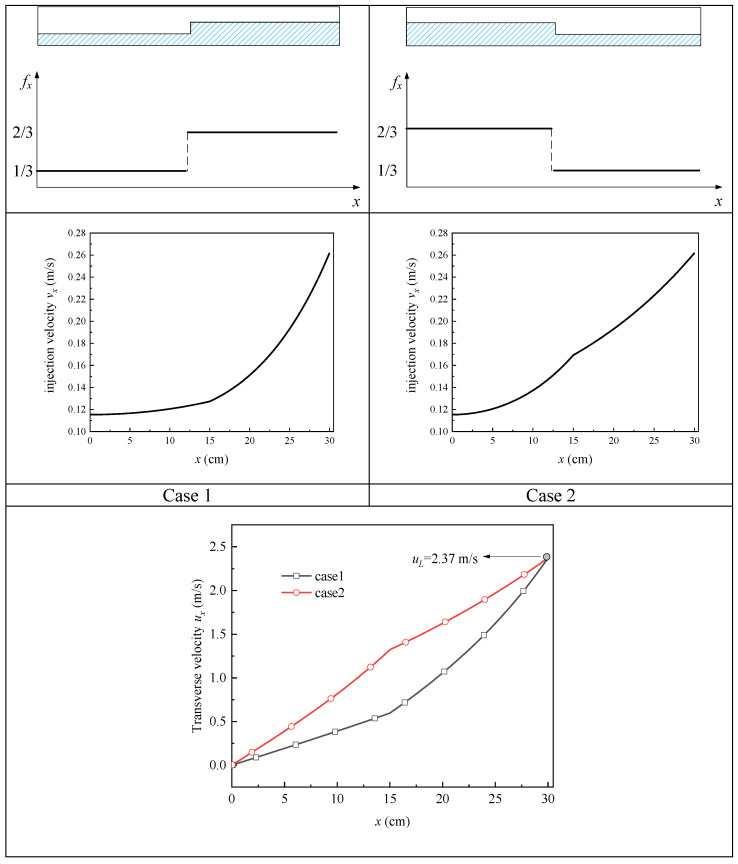
Comparison of cases with two bubbles at different positions.

Furthermore, in order to verify this conclusion, we come to a different situation: the blocked area is divided into two parts (i.e., a large bubble and a small bubble) with their total blocked area is still fixed. In order to simulate this situation, we make the $f_{x}$ distribute as a staircase function, as shown in Figure 8, the “large bubble” blocks 2/3 of the upstream while the “small bubble” blocks 1/3 of the downstream in case 1, with case 2 being the opposite; thus, the value of ($1-\frac{1}{L}\int_{0}^{L}f_{x}dx$) is still kept at 0.5. From the comparison of $u_{x}$ between the two cases in Figure 8, we can see that although these two curves diverge throughout; they intersect at the outlet of the channel, which means that they also get the same $u_{L}$ of 2.37 m/s, although there is a different sorting of the two bubbles. Consequently, it is further verified that the distribution mode of the blockage position at a fixed total percentage of the blocked screen does not affect the outflow rate.

**Figure 9:**
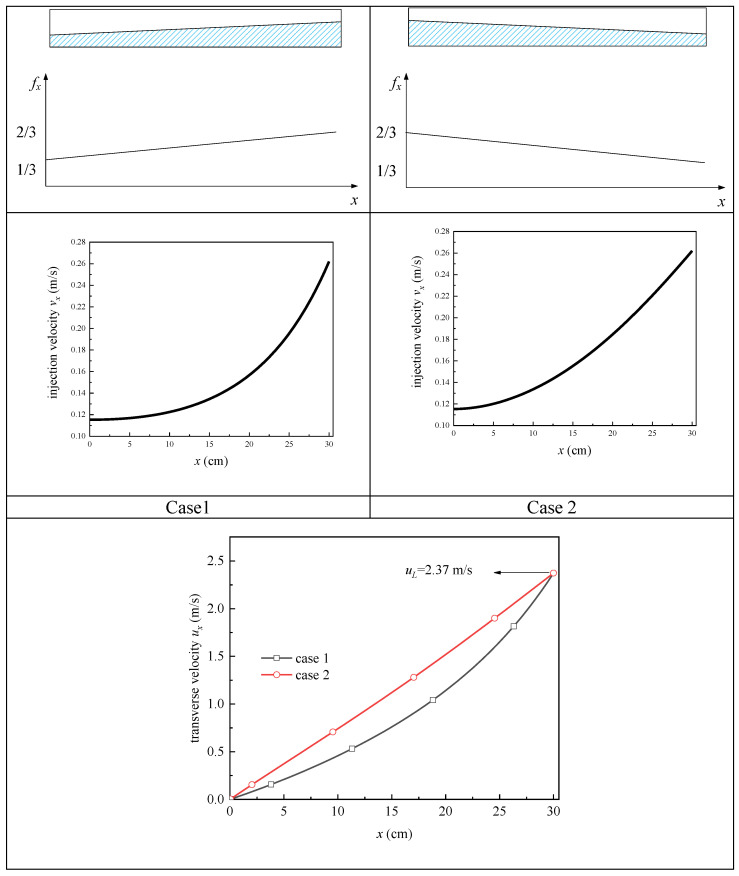
Comparison of cases with a series of bubbles at different positions.

Finally, we come to a more complex situation: the screen is blocked by a series of bubbles of different sizes. As Figure 9 shows, case 1 arranges the bubbles in order from largest to smallest, while case 2 does so in the reverse order. In other words, case 1 undergoes more serious blockage at the upstream, while case 2 is the opposite. The corresponding distribution mode of $f_{x}$ is still kept $1-\frac{1}{L}\int_{0}^{L}f_{x}dx$ as 0.5, with $f_{x}$ changing continuously with *x* direction. Although this difference, the same outflow velocity of 2.37 m/s is still achieved (the curves of transverse velocity merge at the outlet shown in Figure 9).

Overall, the following conclusion can be drawn from the three situations above: as long as the total percentage of the blocked area (i.e., $1-\frac{1}{L}\int_{0}^{L}f_{x}dx$) is fixed, the outflow velocity $u_{L}$ is then fixed, no matter how $f_{x}$ is distributed (being a staircase function or changing continuously). In other words, the blockage position distribution does not affect the outflow rate of a given channel. However, it should be noted that this conclusion is obtained based on assumption 3, that the pressure loss due to wall friction is negligible. In fact, the outflow rate may slightly change at different blockage distributions due to the friction pressure loss (which is proportional to $u_{x}^{2}$), but this deviation could not be significant since the influence of friction resistance has been proved to be slight. Therefore, this conclusion is of great meaning that the design of this kind of LADs could be more flexible without strict requirement of the vapor distribution at any special position. For example, the unimportance of blockage position could give more options for the location of the inlet for pressurizing gas.

#### 3.2.3. Impact of Total Blockage Area

In order to describe how the blockage area affects the outflow rate, we define a “characteristic curve”, letting the blockage rate of the screen (i.e., $1-\frac{1}{L}\int_{0}^{L}f_{x}dx$) be the independent variable and loss rate of outflow velocity (i.e., $1-\frac{{\left(u_{L}\right)}_{blocked}}{{\left(u_{L}\right)}_{ideal}}$) be the dependent variable, where ${\left(u_{L}\right)}_{ideal}$ is the outflow velocity in the case without vapor blockage, and ${\left(u_{L}\right)}_{blocked}$ is the outflow velocity in the vapor-blocked case under the same driving pressure. In fact, the shape of the characteristic curve denotes the robustness of the device against the vapor blockage, which will be discussed in detail below.

The shape of the curve is still influenced by these three factors: driving pressure, the geometric parameters of the channel, and the selection of screens. They will be discussed respectively below.

1. Characteristic curve under different driving pressure

**Figure 10:**
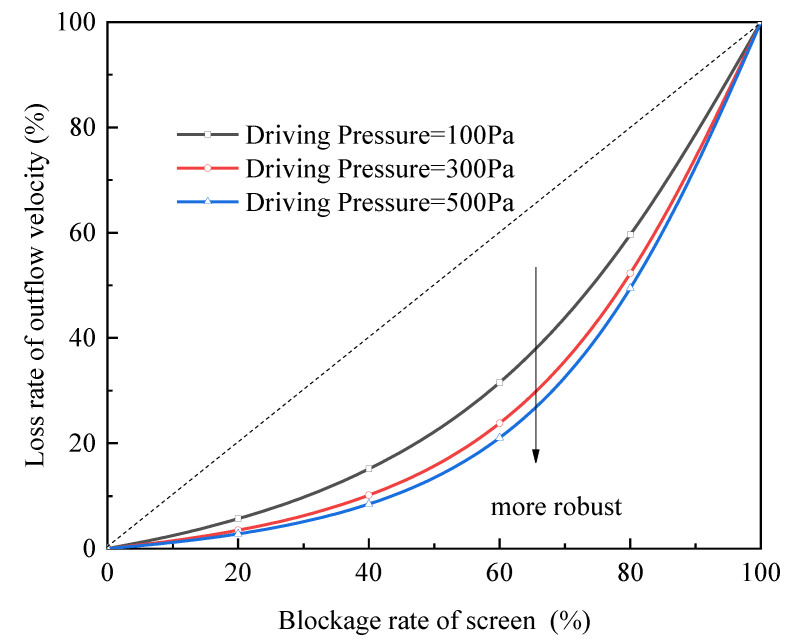
Characteristic curves influenced by driving pressure.

To research the influence of driving pressure on the characteristic curve, three cases driven by different pressure are investigated, while the other conditions are kept the same (i.e., *L* = 30 cm, *H* = 1 cm, with the screen of DTW325 × 2300 is selected). The calculation results are shown in Figure 10, where a curve that bends more downward (along the arrow) represents a more robust performance of a device, because it reflects the device and sustains less loss of outflow rate at the same blockage area. For this reason, higher driving pressure brings about greater robustness (see Figure 10).

Interestingly, all the cases represented by three curves show greater robustness than that by the dashed line (in which the outflow velocity decreases proportionally with the blockage area). To interpret this, when a part of the screen is blocked, other unblocked parts would get higher local injection velocity to compensate for this loss of liquid acquisition rate.

2. Characteristic curve under various *L/H*

**Figure 11:**
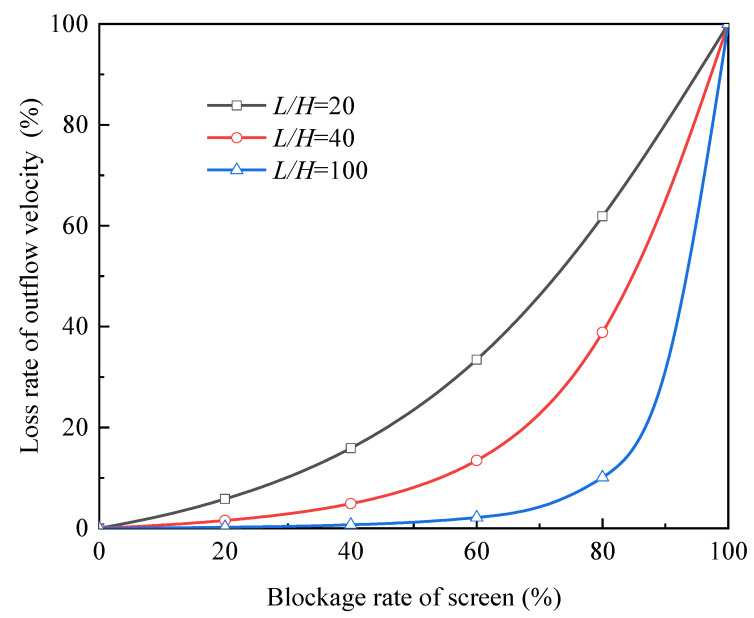
Characteristic curves influenced by *L/H*.

As discussed in Section 3.1.1, the ratio of length to height, *L/H* is the main geometric parameter that affects the outflow velocity. In order to detect how *L/H* influences the shape of the characteristic curve under the vapor-blocked condition, we compare the cases involving three values of *L/H* (see Figure 11), while insisting on selecting the screen of DTW325 × 2300 and driving the device by the pressure of 500 Pa.

Obviously, a greater robustness can be seen under higher *L/H*. In fact, this phenomenon can also be interpreted by Figure 4.: when *L/H* is so high that $u_{L}$ has already approached its upper limit, the decrease of *L/H* will not cause significant loss of $u_{L}$; but when *L/H* is not high enough, $u_{L}$ is sensitive to the change of *L/H*.

3. Characteristic curve under diverse types of screen

**Figure 12:**
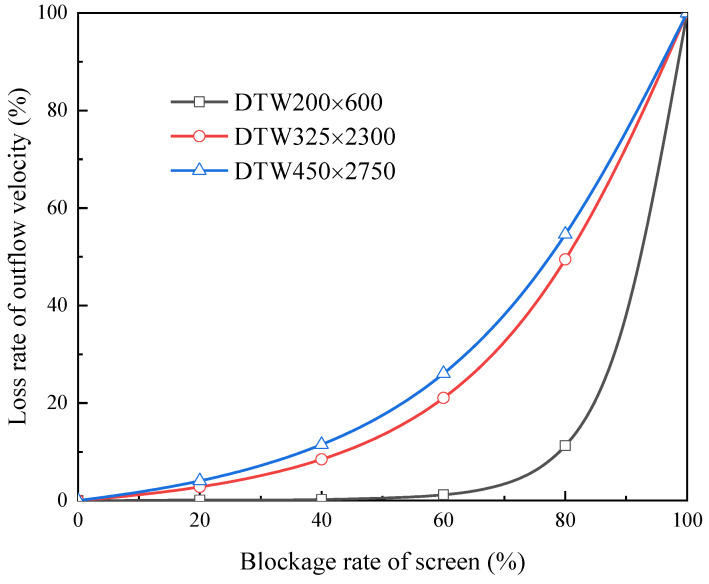
Characteristic curves influenced by the fineness of screen mesh.

As mentioned in Section 3.1.3, the difference between diverse screens mainly lies in the resistance coefficient and bubble point pressure. A coarse mesh screen usually possesses lower resistance coefficients and a lower bubble point pressure. When it comes to robustness, only resistance coefficients (*C_l_* and *C_t_*) are involved. We chose three types of screen for comparison: DTW200 × 600 (*C_l_* = 5.96 × 10^6^, *C_t_* = 15.1), DTW325 × 2300 (*C_l_* = 7.27 × 10^7^, *C_t_* = 65), and DTW450 × 2750(*C_l_* = 1.07 × 10^8^, *C_t_* = 70) [23]. Three identical channels (with the same geometric parameters: *L* = 30 cm, *H* = 1 cm) were equipped with these screens, respectively. The driven pressure was still set at 500 Pa. The resulting characteristic curves are shown in Figure 12, from which higher robustness can be found in coarser mesh screen obviously. In fact, the low resistance of coarser mesh screens makes the channel closer to the upper limit of outflow velocity corresponding to the same driving pressure (i.e., 500 Pa), which brings about greater robustness.

In summary, higher driving pressure, as well as larger *L/H* and coarser screen mesh, would bring about a greater robustness of outflow rate. However, changing these three influential factors to obtain greater robustness is with both advantages and disadvantages. First, driving pressure is strictly up-limited by the value of bubble point pressure minus a safe margin, which means that increasing driving pressure is equal to quitting a part of the safe margin; thus, a tradeoff should be conducted between robustness and safety. Secondly, elevating the length of channel to increase *L/H* would cause the undesired mass gain of the device; hence, designers should keep a balance between robustness and device mass. As for the selection of the screen, the first to be considered for designers is to ensure enough outflow rate without bubble breakthrough failure. Only based on this can robustness can be sought. Since screen mesh can greatly influence the outflow rate (as discussed in Section 3.1.3), designers should be cautious when they decide to adjust the screen type.

## 4. Conclusions

To investigate the effects of vapor-blocked screen on the performance of screen channel LADs during the outflow process, a modified model was established by inserting an “available rate” *f_x_* to analyze the outflow characteristics. The following conclusions can be drawn from this work:

(1) For the cases without vapor blockage, the outflow velocity of the channel is decided by three factors: the geometric parameter of the channel (mainly the ratio of length to height), the driving pressure, and the fineness of screen mesh.
(2) For a given device and under the same driving pressure, as long as the total area of the blocked screen is given, the loss rate of outflow velocity will not change with the changing blockage position. In other words, only the total area of the blocked screen affects the loss rate of outflow velocity.
(3) A “characteristic curve” is proposed to describe the robustness of LAD against screen blockage, letting the blockage percentage of the screen be the independent variable, while the loss rate of outflow velocity is the dependent variable. The device that sustains less loss of outflow rate at the same blockage area performs better robustness.
(4) The three factors could bring about greater robustness: larger ratio of length to height for the channel, higher driving pressure, and coarser mesh of the screen. However, other factors (such as device mass, safe margin, and enough outflow rate) should be also considered comprehensively when adjusting these three influential factors in the search for greater robustness.

## Figures and Tables

**Table 1:** (m/s) of channels with different sizes (screen: DTW325 × 2300; $\Delta P_{d}\;$ = 519 Pa).

	Length	*L* = 5*H*	*L* = 10*H*	*L* = 20*H*	*L* = 50*H*	*L* = 100*H*
Height						
*H* = 1 cm		1.130	1.861	2.429	2.664	2.696
*H* = 2 cm		1.130	1.861	2.429	2.664	2.696
*H* = 3 cm		1.130	1.861	2.429	2.664	2.696
